# Editorial Department

**Published:** 1883-01

**Authors:** 


### The Bistoury.

ELMIRA, N. Y., JANUARY, 1883.

The BISTOURY is published Quarterly, upon the first of April, July, October and January, at Fifty Cents a year in advance.

### Editorial Department.

END OF THE YEAR.—Time to pay subscription for the old and the new year. Many subscribers are even more in arrears. See to it before the sum grows larger.

CHILD MURDER.—A horrible showing is made by Dr. Willis P. King, President of the Missouri State Medical Society, of the work of the 269 professional abortionists whom he declares are practicing their miserable calling in that State. He estimates that over 8,000 children are murdered before they are born, by these fiends. This statement, in itself, is startling enough, but only think of the disease and misery that is inflicted upon the women through this dangerous practice! Since the doctor states that these avowed professional abortionists proclaim themselves as such, publicly, something would seem to be radically wrong with the laws of Missouri.

BODY SNATCHING.—It is greatly to be regretted that so much ado was created in Philadelphia, recently, over the purchase of cadavars by one of the principal medical colleges of that city. It would seem that sufficient unclaimed bodies could be secured from the public hospitals and alms houses of so large a city, with little endeavor, to satisfy reasonable demands for material for dissecting purposes. We doubt whether the professors of the college were in any wise responsible for the grave robberies committed, but that they did not exert a wholesome influence or watchfulness over the "dead house" where their subjects were prepared for the dissection table seems quite probable.

All recognize the necessity for dissection, and perhaps no one can be found who will offer any objection to its legitimate following. But when any institution lends its aid, even by implication, to a system of grave robbery, it is not strange that public indignation is aroused against the dissecting room.

There is a sense of respectful reverence that all of us entertain for our loved dead, that is outraged by the midnight ghoul. It is best that this quality in the human mind should be respected, else might our present salutary laws upon dissection be so modified, by our outraged people, as to seriously interfere with the legitimate and invaluable means of anatomical inquiry.

MEDICAL SOCIETIES.—Much good would come of the monthly meetings of medical men if they would devote less time to trying to convince their fellows how much they know and dwell more upon determining how slender, after all, their abilities are. In perusing many of the papers, written for what would seem to be to portray the writers as very learned men, and particularly directed to the non-professional reader (with the same aim vividly in view), very little of real value to the profession is ever elicited. If the proceedings of such societies were less conspicuously advertised in the secular press, one incentive for the compilation of very many of these so-called papers would be removed.

We much admire the plan adopted by the "Medical Society of the County of Albany," in this State, the members of which are really scientific men, who, in writing papers, have something to communicate to the members, often of the most absorbing interest to the profession. That being the case, they do not publish them in the newspaper, but in pamphlet form for the sole benefit of their professional brethren. Their journal is named "*The Medical Annals*," and is devoted almost entirely to the proceedings of the society named.

We heartily commend the undertaking, and recommend the plan to other societies as being at once the most modest and satisfactory method of preserving, in printed form, the proceedings of scientific societies.

ABDOMINAL SURGERY.—Year by year does the surgeon grow in skillfulness and in his capability for relieving human ailments. Heroic operations are performed at this day—and with marvelous success—that, but a few years ago, would have been considered but little short of murderous in design. Recent operations have been performed wherein portions of diseased liver have been removed and the patient cured of the ailment for which the operation was made. One kidney has been successfully removed, for incurable disease of that organ. Part of the stomach has been excised for cancerous disease. The ovaries have been so frequently removed of late years, that the operation for "ovariotomy" is looked upon as one of the most successful of procedures. Portions of the intestines have frequently been excised, their cut ends brought together and securely healed. Operations are upon record where the gall-bladder has been opened, a pint or more of gallstones extracted and others removed from the ducts. All of the abdominal organs have been invaded by the surgeon's knife and the patients restored to health and usefulness. The ingenious instruments and unique methods by which these operations are made possible, are no less wonderful than the results from the operative procedure. What is equally as gratifying, and which appeals to our sense of patriotism, is the fact that most of these brilliant operations have been performed by the eminent surgeons of our own country.

---

ALWAYS A LITTLE SICK.—It has been noticed, and the fact much commented upon, that the serious and fatal sicknesses that have transpired among adults in our own city this winter, selected victims from among our heartiest and most robust people.

"What, he dead?" exclaimed a dyspeptic and correspondingly anæmic individual on the street one day. "Why he was such a large, stout, ruddy-looking fellow—I thought he'd outlive me at least fifty years!"

Yes, so he was. But all these attributes of seeming or real healthfulness is but slender protection against disease, unless the additional safeguard—expressively portrayed in the usual sentence—"take care of yourself, old fellow," is faithfully employed.

It will be noticed that those men who are always just a little sick—grunting and complaining of headaches, attacks of indigestion and the like, usually live to a good old age, acting as pall bearers to their more healthful, robust, jolly friends, who eat unsparingly at the club suppers, smoke a dozen cigars daily, and enjoy a champaigne punch at bed time. These hearty fellows who *dare* do all this, pride themselves upon such ability, while they look with pity upon the chap who abstains from all these delightful luxuries. This abstemiousness may not be from choice—from a wise conclusion that it does not lead to good health; but simply because he can not so indulge without suffering from a headache the following day. He would *like* to eat and drink, perhaps, but can not afford the certain bad effects to follow, so he becomes careful—eats abstemiously, healthfully, and upon proper occasions. All this care of himself is exercised because he is one of those who is always "a little sick." Such a condition of health is not to be despised. It is a proper and needful safeguard to its possessor. It teaches him at all times, to consider his health first, while he recognizes the evils that will surely follow unusual indulgences in eating and drinking.

The man of weak digestion, the dyspeptic, the sufferer from headache, etc., is not to be commiserated; rather is he to be congratulated, since his slight infirmities are constant reminders to be constantly on guard, so defeating the attacks of more serious ailments.

---

CHRISTMAS AGAIN.—For the twentieth time we will pen a Christmas greeting to our subscribers. Of course, there is nothing new to be written. Although twenty years older, we can not see that any of our enthusiasm for the festivities of the holidays is lacking. We like to see the young folks making preparations for surprising father and mother with samples of their handiwork—tokens of affection that seem to please the giver more than the receiver. We rejoice in the collecting together of the scattered members of the family, who seek the good old homestead and are once more collected about the family table, recounting the delights and successes of the year. And the little ones—the happy, merry children, with faith still unshaken in the Christmas visit of dear old Santa Claus. How we love to listen to their innocent prattle, hear their expressed hopes for what the mysterious visitor may bring them. Their smiling faces, sparkling eyes, joyous shouts, while they count the days that Santa Claus is still distant from them, makes even an old fellow watch for the day with something of childish glee.

The holiday season should be, and doubtless is, the happiest season of all the year. Many, very many are the hearts gladdened with its coming—few, we hope, that are filled with grief. The BISTOURY sends to all its greeting, wishing its readers a Merry Christmas, a happy, prosperous New Year.

THE ADVANTAGES AND DISADVANTAGES that crop out and arrange themselves antagonistically in the daily, yea, hourly experience of one whose wife has gone visiting, might be entertainingly portrayed. So quietly and unobservedly are her household cares performed, that the male members thereof are not really aware of the manifold conveniences she arranges for their comfort, while they are about their daily avocations. But let her desert the family dwelling for a week, then are her ministrations negatively made prominent. A fellow then seems to be steering through the week without compass or chronometer. He don't know when clean-shirt day arrives, or if he is reminded of it by the soiled appearance of collar and cuff, he does not feel equal to the perplexity of searching for one, much less preparing it with the necessary studs and cuff buttons. The procedure is vexatious, and consumes so much time that a deal of sympathy is due the chap who is found with soiled or neglected garments, when it is known his wife is from home. Neither is it conducive to pleasant reflections nor provocative of dignified expletives to find, when searching in the drawer set apart for your underwear, that they are not constructed upon the plan best suited to your form; but, contrarywise, of a pattern that would, or should, mystify an unmarried gentleman, to say nothing of the great inconvenience that would surely overtake one in trying to wear them. This ill-assorting of garments could only occur through the stupidity of the maid in the absence of her mistress—another count for the disadvantages of the situation. We have also noted that a man is not calculated for preparing the little people for school—the boys are easily enough constructed—but when it comes to bedecking the girls with bonnets that have no special mark indicating the front; aprons that puzzle you to determine whether to be buttoned front or rear; collars with fantastic cords and tassels that may be intended to encircle the waist—to hold the *tout ensemble* on—but as to the use of, or place for, you are in doubt; all these situations are highly perplexing and lead you to mentally inquire *when* the wife is likely to return. These are, by no means, all the inconveniences that occur to the man with an absent mate—but will answer to illustrate such a chap's deplorable situation. The only consolation or comfort that can possibly be gleaned from such a desertion is that, having seated yourself at the microscope and there finding a new rotifer, you can observe him to your heart's content—during the entire night, if you will—with no fear of some one gliding softly to your side, turning out the light, and in the gentlest and blandest of tones informing you—"Come, it has long since been your bed-time, dear!" Or, bolstered up in bed, with conveniently arranged light, and entertaining book, no one fidgets at your side, overturning book and knocking spectacles from your nose in an endeavor to shield her face from the light with peculiar and harassing arrangements of the bedclothes. These be two of the advantages of the situation—and include, now that we reflect upon it, about all that we can recall.

Summing up, then, we are led reluctantly to the definite conclusion that a fellow's wife should never go visiting; and we are in favor of an amendment or addendum to the civil-service reform bill that shall forever prohibit her from so doing.

---

BLOOD CORPUSCLES.—It is not generally understood by the laity, that any difference prevails between the blood corpuscles of man and those of animals, birds or reptiles. Blood is looked upon as blood, whether it comes from the creatures named, or from the circulation of man. But when an individual, suspected of having committed murder, accounts for the presence of blood upon his clothing, by declaring that it is due to his having killed a chicken, in the decapitation of which the blood spurted upon his trousers, the microscope can be brought to bear upon the stain and at once reveal whether his statement is correct or not.

Such a case recently occurred in Evansville, Ind., where Dr. J. O. Stillson, an expert microscopist, convinced the jury that the blood claimed to be from a chicken, really was human blood. The manner in which such determination was arrived at may interest our readers. It is as follows: The blood found upon the clothing is moistened with a solution of chloride of sodium (common salt) when the dried corpuscles are restored to their normal condition. They are then smeared upon a piece of glass, a very thin circle of glass laid over them, when the corpuscles are examined under the microscope. A power of from four to six hundred diameters is used for the purpose. If the specimen be from man, the corpuscles will appear round, with a bright center, due to reflection of light from its concave surface. In the case of all birds, the corpuscles will be distinctly oval in form, in size far exceeding that of man, with a well-defined neucleus. Other differences could be pointed out, but their greater size, oval shape and central nucleus is sufficient to illustrate very clearly the decided difference between the corpuscles of man and that of any bird.

Some times the blood corpuscles are double stained (by colors adapted to that sort of work) when the peculiarities of the corpuscles are more clearly brought out. The human corpuscles absorb but one of the stains, while that of the bird will appear blue, in the protoplasmic portion surrounding the nulceus, and the nucleus will take on the green color.

The blood of some animals is so nearly like that of man, that they can only be distinguished by very carefully conducted micrometric measurements.

Reptiles have such enormous corpuscles—all oval in shape—with prominent nucleus, that they are quite easily recognized. The microscope has become a very valuable aid in detecting criminals in the manner described. Asserting blood to be from animals or birds, when really from man, will not pass without a challenge and complete contradiction in these days of the perfected microscope.

---

TELEPHONE VEXATIONS.—The greater the conveniences with which men are surrounded, enabling them to conduct their business more speedily, and therefore more profitably, the greater and more clamorous is their demand for the thorough working of all such appliances. The telephone is a very great convenience. It is also exceedingly vexatious. Just why the latter attribute is present, we do not know—we have not visited the exchange to ascertain. But we *do* know, and have had abundant opportunities for verifying the experience, that the service at said exchange is not what it should be. Whether the attendants there are at fault, whether there are not enough of them, whether too many telephones are working on the same line, or whether all these reasons combine to make the service very faulty, does not appear. Certain it is, however, that subscribers to a very costly luxury should not be subjected to the great annoyance of repeatedly calling for the exchange, and too often without receiving any response. It has become too frequently the case that one would prefer to walk or send to the point with which communication is desired, rather than run the risk of securing the exchange the first two or three times a call is made. As declared in the beginning, the greater the convenience the least cause for complaint is expected of it. The telephone service, if conducted with that carefulness that subscribers have a right to expect of it, and with that faultlessness of which it is reasonably capable, would be one of the greatest conveniences and labor-saving devices yet given us. Let it be improved.

---

THE OFFICE OF CORONER.—A thousand years ago, long before the multiplicity of laws permitted of the escape of criminals upon purely technical grounds, England instituted the office of Coronor. From the mother country, therefore, was borrowed the idea that a special officer, with a jury of six stupid (usually) men, should determine whether any real *cause* existed for a man to die from apoplexy, "heart disease," a pistol ball penetrating the brain, or a six-inch knife blade thrust through the heart. Indeed, our attention was recently called to a case where a drunken man had tumbled from the platform of a railroad car, three cars having passed over his body, after which his brains were gathered up a mile from where his head was found, a hawk shot in an endeavor to carry away the lungs of the somewhat mangled body, when, having gathered up the remains, it was thought necessary that a Coronor and jury be employed, for six days, to determine what that man died of, and, if dead, what killed him—all this at a cost to the county of more than one hundred dollars.

We suppose that the office of Coroner was originally created for the purpose of having a skilled physician, familiar with the usual causes of death, determine whether, in cases where death was sudden, or surrounded by suspicious circumstances, it was worth while to enter into a costly investigation of the causes that led to the death. But, at this day, it has been converted into a profitable employment, whereby a Coroner can reap two thousand dollars a year, if he is sufficiently diligent in hunting up cases of accidental or sudden deaths arising from natural causes. We do not find so much fault with the Coroner for following up the perquisites of his office, as we do with the foolish and quite unnecessary law that affords him the opportunity.

In France and Germany—we are creditably informed—no such office as Coroner exists. Deaths, arising from suspicious causes, are investigated by officers corresponding to our Sheriffs. And why not, pray? Why should not the Sheriff employ an expert physician to hold a post mortem examination, as is now practiced by all our Coroners? What use or necessity exists for such an officer, particularly when he calls in expensive professional aid where himself was expected to act? As well can our Sheriff do that, an officer sufficiently salaried to perform the duties of the Coroner quite as well, and perhaps with less incentive for conducting investigations where they are manifestly not needed.

In looking over the proceedings of the Supervisors of the county for the past year, we find that an unusual number of inquests, upon dead bodies, were held. The cost to the county for conducting these examinations, post mortem, and otherwise, is very great. In carefully examining the list, we cannot find a solitary case where a Coroner's inquest was necessary to determine the cause of death, nor were there the slightest indications that death was from other than entirely natural causes. It is safe to presume that, had not the office of Coroner existed, no call would have been made, by any citizen, to have any one of these cases investigated—with a view of unearthing criminality in the death cause.

What is true of our own city, doubtless is equally so of others. The larger the population of the county the fatter are the emoluments of the office. Now, can not the office of Coroner be abolished, with its duties delegated to the Sheriff of the county? If not, *why* not? As a matter of economy, certainly, it would be a change greatly to the advantage of tax-payers, without in the least defeating or crippling the ends of justice, as applied to murders or those suspected of such criminality.

NATIONAL BOARD OF HEATH.—We see it stated, in our medical exchanges, that this very useful organization is likely to die from lack of an appropriation from Congress to enable it to continue its work. We hope our legislators will not commit so great a blunder. The board has done most excellent service throughout the country in establishing inspectors of small-pox and other infectious diseases. Has instituted inspectors for incoming steamboats. Has been conducting costly scientific investigations into the nature of diphtheria, malaria and soil pollution. These investigations should be continued.

